# Herpes simplex virus type 2 infection increases human immunodeficiency virus type 1 entry into human primary macrophages

**DOI:** 10.1186/1743-422X-8-166

**Published:** 2011-04-12

**Authors:** Elena Sartori, Arianna Calistri, Cristiano Salata, Claudia Del Vecchio, Giorgio Palù, Cristina Parolin

**Affiliations:** 1 Department of Histology, Microbiology and Medical Biotechnologies, Division of Microbiology and Virology, University of Padova, Via A. Gabelli 63, Padova 35121, Italy; 2 Department of Biology, University of Padova, Via U. Bassi 58/b, 35131 Padova, Italy

## Abstract

Epidemiological and clinical data indicate that genital ulcer disease (GUD) pathogens are associated with an increased risk of human immunodeficiency virus type 1 (HIV-1) acquisition and/or transmission. Among them, genital herpes simplex virus type 2 (HSV-2) seems to play a relevant role. Indeed, the ability of HSV-2 to induce massive infiltration at the genital level of cells which are potential targets for HIV-1 infection may represent one of the mechanisms involved in this process. Here we show that infection of human primary macrophages (MDMs) by HSV-2 results in an increase of CCR5 expression levels on cell surface and allows higher efficiency of MDMs to support entry of R5 HIV-1 strains. This finding could strengthen, at the molecular level, the evidence linking HSV-2 infection to an increased susceptibility to HIV-1 acquisition.

## Findings

Herpes simplex virus (HSV), and especially HSV type 2, represents one of the most widely spread pathogen causing genital ulcer disease (GUD). Different studies have associated GUD aetiological agents in general, and HSV-2 in particular, with a higher risk to acquire and/or transmit HIV-1 infection [1-3]. A number of biological and molecular factors may explain this evidence. Both the physical disruption of the epithelial/mucosal barrier and the cellular inflammatory response characterizing GUD could facilitate HIV-1 acquisition, by providing the virus with access to a large number of CD4-positive cells. Moreover, several *in vitro *studies have underlined molecular mechanisms by which HSV can directly influence the HIV life cycle in HSV-HIV coinfected cells [2,4]. Finally, randomised controlled trials have been conducted in coinfected individuals to evaluate the effect of HSV-2 suppressive therapy on HIV-1 genital shedding and plasma HIV-1 RNA, showing, in most cases, a negative impact on HIV-1 replication [5-7]. A recent study conducted by Zhu and co-workers [8] showed a persistence of HIV receptor-positive cells in genital skin after HSV reactivation. In the genital tract, macrophages represent one of the main target of HIV-1, especially during primary infection. In this study we wanted to analyze the ability of HSV-2 to infect human macrophages and to influence HIV-1 super-infection.

**Figure 1:**
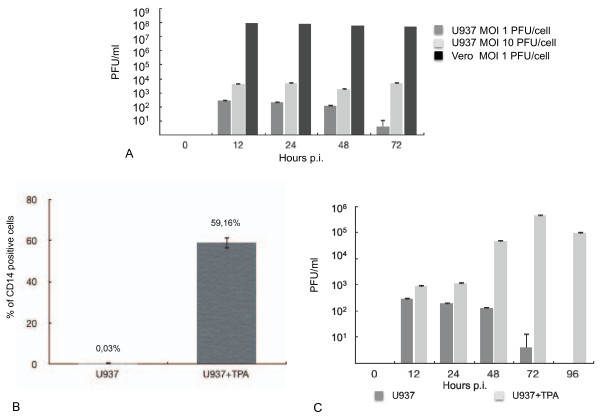
**HSV-2 ability to infect human monocyte cell line U937 relies on their differentiation level**. A) HSV-2 replication in undifferentiated U937 cells. The U937 cell line was infected with two different MOI of HSV-2, as reported in the graph legend. Vero cells were infected as a control. HSV-2 titre in the cellular supernatants was measured by plaque assay, at different times post-infection (p.i.). B) Effect of TPA treatment of U937 cells on CD14 expression. The effect of TPA treatment on U937 cell differentiation state was analyzed by CD14 FACS analysis. The percentage of positive cells is reported. C) HSV-2 replication in TPA-treated U937 cells. Undifferentiated or TPA-treated U937 cells were infected with HSV-2 (MOI of 1 PFU/cell). HSV-2 titre in the cellular supernatant was measured by plaque assay. In all cases, the reported values represent the mean of four independent experiments. The error bars represent the standard deviation.

Firstly, we selected the human monocyte U937 cell line (ATCC^® ^Number CRL-1593.2) as experimental set and we infected them with HSV-2, strain G (kindly provided by Dr. Peggy Marconi, University of Ferrara, Italy). Briefly, the virus was grown and titrated by plaque assay on African green monkey kidney cells (Vero), as previously described [9]. U937 cells (1 × 10^**6**^) were infected with HSV-2 at two different multiplicity of infection (MOI of 1 and 10 plaque forming unit, PFU, per cell). Cells were left in contact with the virus for two hours at 37°C and, after three washing with phosphate buffered saline (PBS), cultured in Roswell Park Memorial Institute medium (RPMI 1640), with addition of 10% heat-inactivated foetal bovine serum (complete medium). HSV-2 replication was followed by titration of the virus released in the cellular supernatant. In contrast with fully permissive Vero cells, our data show that U937 cells do not support a significant HSV-2 replication and that, at least in the case of the MOI of 1 PFU/cell, the viral titre declines over time (Figure 1A). It has been previously reported that monocytes display an intrinsic resistance to HSV type 1 (HSV-1) infection, depending on the cellular differentiation level along the monocytic pathway into functionally and morphologically mature non-proliferating cells, that can be achieved by 12-O-tetradecanoylphorbol-13-acetate (TPA) treatment [10-12].

Thus, in order to analyze whether the low susceptibility to HSV-2 infection displayed by U937 cells could be related to their differentiation level, the cells were induced to differentiate by treatment with TPA (50 ng/ml). After twelve hours of incubation, U937 cells were washed twice with PBS and cultured for additional twenty-four hours in TPA-free medium, in order to avoid possible effects of residual TPA. The percentage of cells positive for CD14 surface expression, a marker of macrophage differentiation [13], was then determined by Fluorescence-Activated Cell Sorting (FACS). Briefly, 1 × 10^6 ^cells were harvested and directly incubated for one hour in cold PBS containing 1:100 (v/v) of an anti-human CD14 primary antibody (Li StarFISH). A fluorescein isothiocyanate (FITC)-conjugated anti-rabbit immunoglobulin G antibody (Santa Cruz) was employed as secondary antibody and the fluorescence was evaluated by FACS analysis (FACScalibur, Beckton Dickinson). As reported in Figure 1B, after TPA treatment the percentage of CD14-positive U937 cells is significantly increased. Differentiated U937 cells were infected with HSV-2 (MOI of 1 PFU/cell). Infectious virus yields, which peaked approximately three days post-infection, appear to be significantly higher than those obtained from undifferentiated U937 cells (Figure 1C). Thus, our data suggest that HSV-2 replication efficiency is dependent on the differentiated phenotype of U937 cells along the monocytic pathway. Interestingly, while untreated U937 cells did not display a significant HSV-2 induced cytopathic effect (CPE), TPA-differentiated U937 cells were fully susceptible to viral CPE (data not shown).

**Figure 2:**
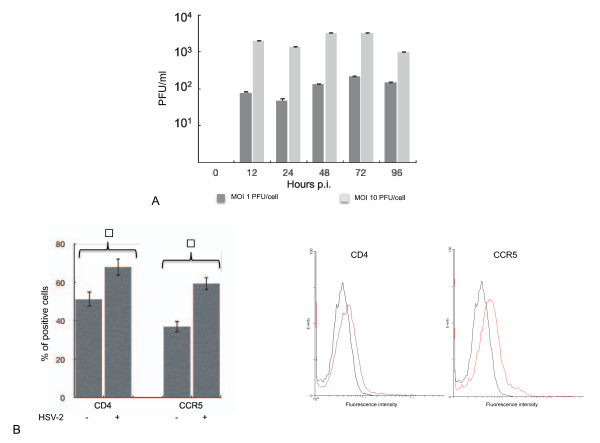
**HSV-2 infects human MDMs and alters CD4 and CCR5 expression on the cell surface**. A) HSV-2 replication in human MDMs. Purified human MDMs were infected with two different MOI of HSV-2, as indicated. HSV-2 titre in the cellular supernatants was measured by plaque assay at different times post infection (p.i.). B) Effect of HSV-2 infection on CD4 and CCR5. Uninfected MDMs (HSV-2 -) and HSV-2 infected MDMs (HSV-2 +) were analyzed for CD4 and CCR5 cells surface expression by FACS analysis, 72 hours post infection. The percentage of CD4 and CCR5 positive cells is reported (left panel). The right panel reports the FACS histograms showing the mean fluorescence intensity for both CD4 and CCR5 molecules in uninfected (black line) and HSV-2 infected (red line) MDMs. When values are reported, they represent the mean of four independent experiments. The error bars represent the standard deviation. Statistically significant differences between HSV-2 infected and un-infected cells, identified by employing the unpaired t-Student test, are indicated with the star (p < 0.05).

Next, infection of primary monocyte-derived macrophages (MDMs) was performed. MDMs were obtained from at least three pooled buffy coats of HIV-1 seronegative healthy blood donors, by Ficoll-Hystopaque gradient, followed by plastic adherence of PBMCs for sixteen hours in complete RPMI. Non-adherent cells were removed, and adherent cells were extensively washed with PBS and grown in complete RPMI supplemented with Granulocyte-Macrophage Colony-Stimulating Factor (GM-CSF, 500 U/ml). The cells were cultured for one week, before evaluating the preparation purity by measuring the percentage of CD14-positive cells through FACS analysis, as described above. The cut-off employed to accept the purity of MDM preparation was a CD14-positive percentage higher than 90% [13]. Purified MDMs (2 × 10^6^) were infected with HSV-2 strain G at two different MOIs, 10 PFU/cell and 1 PFU/cell, following the same experimental procedure described for the U937 cell line. After viral adsorption, the infected MDMs were maintained in complete RMPI medium supplemented with GM-CSF. Our data show that MDMs support HSV-2 replication. As expected, at the MOI of 10 PFU/cell the infection appears to be more efficient, achieving a peak in the viral titre seventy-two hours post-infection (Figure 2A). No clear viral induced CPE could be observed at any time post-infection (data not shown). Next, we analyzed the effect of HSV-2 infection on HIV-1 receptor and coreceptors expression on macrophage cell surface. Indeed, HIV-1 entry into target cells involves the interaction of the viral envelope glycoproteins with the host cell receptors, CD4, and one of two coreceptors CCR5 or CXCR4. The chemokine receptors CXCR4 and CCR5 represent indeed the coreceptors for T-cell-tropic (X4-tropic) and macrophage-tropic HIV-1 (R5-tropic) strains, respectively. Dual/mixed-tropic populations are also present in HIV infected patients and evidence has been collected in antiretroviral-naive patients suggesting that the majority of viruses within these dual/mixed-tropic populations use CCR5 [14]. Moreover, natural history studies of HIV-1 infection have shown that most patients harbour R5-tropic virus populations soon after infection and through the asymptomatic phase [15,16]. Thus, R5-tropic strains appear to be the most relevant during HIV-1 acquisition and early phases of infection. Taking into account all these considerations, we focused our attention on HIV-1 CD4 receptor and on the CCR5 coreceptor. 2 × 10^6 ^purified MDMs were infected with HSV-2 at the MOI of 10 PFU/cells and, seventy-two hours later, the cells were harvested and analyzed by FACS for the level of CD4 and CCR5 surface expression. In particular, either a CD4 (1:200 v/v, BD Pharmingen) or a CCR5 (1:50 v/v, BD Pharmingen) specific primary antibody, along with a FITC-conjugated anti-rabbit immunoglobulin G secondary antibody (Santa Cruz), were employed on fresh MDMs. Our data show that the percentage of both CD4 and CCR5 positive cells increases after HSV-2 infection (Figure 2B) (p < 0.05). At the same time, also the expression levels of HIV-1 receptor and coreceptor on the cell surface is slightly enhanced, as indicated by the shift in the mean fluorescence intensity displayed by the FACS histograms (Figure 2C).

In order to analyze whether the HSV-2 effect on CCR5 expression may have an impact on HIV-1 ability to enter macrophages, we employed a modified version of the previously described *env*-complementation assay, in which the HIV-1 envelope glycoprotein, expressed *in trans*, complements a single round of replication of an *env*-deleted provirus expressing the chloramphenicol acetyltransferase (CAT) gene [17,18]. Since the defective HIV is capable of only one cycle of replication, this complementation assay allows us to quantitatively measure the abilities of the cells to support the entry of HIV-1 variants containing different envelope glycoproteins, by evaluating the level of CAT expression in the target cells. This assay represents an invaluable tool to dissect the contribution of viral/cellular determinants involved in HIV-1 entry [17,18]. Recombinant HIV-1 viruses were produced by cotransfection of human embryonic kidney cells (293T, ATCC^® ^Number: CRL-11268TM) with two plasmids, pSVCvpr^+^vpu^+^nef^+^Δenv-CAT and pSVIIIenv. The pSVCvpr^+^vpu^+^nef^+^Δenv-CAT is a derivative of the pSVC21, containing the HIV-1 HXBc2 molecular clone [19], where the *vpu, vpr *and *nef *sequences were substituted with those derived from the pNL4-3 (*vpu/vpr*) [20] and pLAI (*nef*) [21] molecular clones, in order to introduce functional *vpu, vpr *and *nef *genes. Starting from the pSVCvpr^+^vpu^+^nef^+^, we introduced by molecular biology techniques a 580 bp deletion (nucleotides 7041-8621) in the *env *gene and cloned the chloramphenicol acetyltransferase (CAT), obtained from the v653 RtatC vector [22], at the *Bam*HI site (nucleotide 8053) [19]. The CAT gene is under the transcriptional control of the HIV-1 LTR and is expressed from a subgenomic mRNA generated by the same splicing events used for the natural HIV-1 *nef *message. Different pSVIIIenv plasmids encoding the HIV-1 Rev protein along with the envelope glycoproteins derived from laboratory-adapted T-cell-tropic (HXBc2), macrophage-tropic (JRF-L and ADA) and primary dualtropic (89.6) HIV-1 isolates, which can use CXCR-4 [23], CCR5 [24] or either one [25] respectively, as a coreceptor, were adopted. Since the viral proteins are expressed in a context similar to that occurring in the authentic provirus, the levels of gene expression achieved are expected to resemble those in HIV-1-infected cells. Briefly, 293T cells were cotransfected by the calcium phosphate method with 20 μg of the pSVCvpr^+^vpu^+^nef^+^Δenv-CAT plasmid and 5 μg of pSVIIIenv plasmids expressing the HIV-1 HXBc2, ADA, JRF-L, or 89.6 envelope glycoproteins to produce recombinant virions. Control viruses lacking envelope glycoproteins were produced by transfecting 293T cells with the pSVCvpr^+^vpu^+^nef^+^Δenv-CAT plasmid alone.

**Figure 3:**
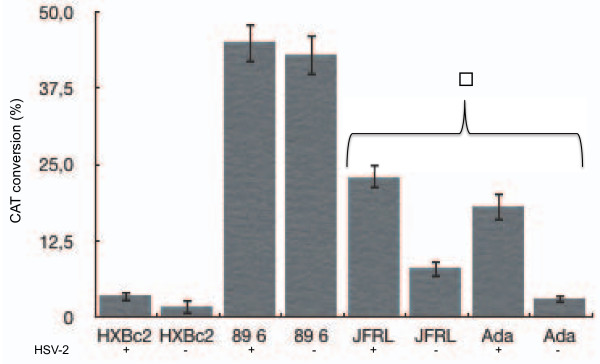
**Effect of HSV-2 infection on HIV-1 entry into MDMs**. Single round replication HIV-1 recombinant particles pseudotyped by different envelopes (HIV-env) were employed to transduce uninfected (HSV-2 -) or infected (HSV-2 +) MDMs, 72 hours post HSV-2 infection. The entry into cells was analyzed by CAT assay performed on the cell lysates, normalized for protein content. The percentage of CAT conversion is reported and represents the mean of four independent experiments. The error bars represent the standard deviation. Statistically significant differences between HSV-2 infected and un-infected cells, identified by employing the unpaired t-Student test, are indicated with the star (p < 0.05).

Twelve hours post-transfection, 293T cells were washed and cultured in RPMI supplemented with 10% FBS. Conditioned medium containing recombinant viruses was harvested and filtered (0.45-μm-pore-size filter) twenty-four hours later. Recombinant viral particles were quantified by reverse transcription (RT) assay. Briefly, virions were precipitated from 1 ml of the filtered supernatants by centrifugation at 13,000 rpm for sixty minutes at 4°C. The precipitate was resuspended in 10 μl of a buffer containing 50 mM Tris-HCl pH 7.5, 1 mM dithiothreitol (DTT), 20% glycerol, 250 mM KCl and 0.25% (v/v) Triton X-100, transferred in dry ice and lysed through three cycles of freezing and thawing. The sample was added to a reaction mixture containing 50 mM Tris-HCl pH 7.5, 7.5 mM MgCl_2_, 0.05% (v/v) Triton X-100, 5 mM DTT, 100 μg/ml polyA, 10 μg/ml oligo-dT and 2 μCi of ^3^H-dTTP (43 Ci/mmole) in a final volume of 50 μl. The reaction was incubated for one hour at 37°C and then transferred on Whatman filters. Filters were immediately washed three times in SSC 2× (0.3 M NaCl, 0.03 M sodium citrate pH 7.2) for 10 minutes each, twice in absolute ethanol for ten seconds each and then dried. The radioactivity was measured by using a scintillator (Rackbeta 1214 Wallac) and expressed in counts per million (cpm). In parallel, 1.5 × 10^6 ^of purified MDMs were cultured in complete RPMI containing GM-CSF in six-well plates for one week, before being infected with HSV-2 at the MOI of 10 PFU/cell, as previously described. Seventy-two hours later, the cells were transduced with 100,000 H^3 ^cpm RT units of the different HIV-1 recombinant particles previously generated and expressing the CAT reporter gene. Seventy-two hours post-transduction, the MDMs were harvested, lysed in 150 μl of 250 mM Tris-HCl pH 7.5 and then assayed for CAT activity, as previously described [22]. The different forms of acetylated chloramphenicol were separated by thin layer chromatography (TLC) and visualized with an autoradiografic exposure of twelve hours (Kodak Biomax films). The quantitative evaluation was obtained by cutting the TLC paper at the level of the corresponding spots, and by performing a quantification of the spots at the scintillator. The percentage of conversion in the acetylated forms was calculated as follows:

% of conversion = (mono- + di-acetylated forms)/(non acetylated + mono- + di-acetylated forms). Calculated with the above formula, the percentage of conversion is linear for values up to 50%. Results are reported in Figure 3. As expected, the X4-tropic virus (HXBc2 *env*) does not infect efficiently MDMs, while the dual tropic strain (89.6 *env*) displays the higher efficiency of infection in all the conditions tested. Our data demonstrate that, following HSV-2 infection, the ability of HIV-1 to superinfect target cells is overall enhanced. The effect is statistically significant (p < 0.05) when R5-tropic recombinant viral particles are examined, correlating this observation, at least partially, with the HSV-2 related increase in CCR5 expression levels on MDMs surface. Not surprisingly [25], the 89.6 dual tropic strain was extremely efficient in macrophage infection and the observed slight increase in HIV-1 entry upon HSV-2 infection (Figure 3) was not statistically significant (p > 0.05).

As mentioned above, interaction between HIV-1 and other sexually transmitted disease pathogens has been a subject of extensive investigation [1-9]. In particular, HSV-2 by affecting genital mucosa integrity and the function of cells physiologically forming a barrier against HIV-1 infection, such as Langherans cells [26], alters the cellular environment at the portal entry and facilitates HIV-1 acquisition/transmission [27]. Significantly, in this study we demonstrate that HSV-2 infected macrophages, which represent one of the main target for HIV-1 infection at the genital mucosal site, display an enhanced expression of HIV-1 CCR5 coreceptor. This feature renders the cells more susceptible to infection especially by R5-tropic HIV-1 strains, which play a significant role in primary infection [14-16]. Human macrophages constitutively express HSV receptor HVEM (herpesvirus entry mediator) [28] and can be infected by HSV-2 [28, this report]. Taking into account this evidence and *in vivo *data demonstrating the enrichment of HIV receptor-positive inflammatory cells in the HSV-2 positive patient genitalia [8], our results describe one of the possible molecular mechanisms by which genital herpes may facilitate HIV-1 acquisition in HSV-2/HIV-1 coinfected patients.

## Competing interests

The authors declare that they have no competing interests

## Authors' contributions

Conceives and designed the experiments: ES, AC, GP, CP. Performed experiments: ES, CDV, CS. Analyzed the data: ES, AC, CS, CDV, GP, CP. Wrote the paper: AC, CP. All authors read and approved the final manuscript.
